# Exploring the views of female genital mutilation survivors, their male partners and healthcare professionals on the timing of deinfibulation surgery and NHS FGM care provision (the FGM Sister Study): protocol for a qualitative study

**DOI:** 10.1136/bmjopen-2019-034140

**Published:** 2019-10-17

**Authors:** Laura Jones, Emma Danks, Joanne Clarke, Lailah Alidu, Benjamin Costello, Kate Jolly, Alison Byrne, Meg Fassam-Wright, Pallavi Latthe, Julie Taylor

**Affiliations:** 1 Institute of Applied Health Research, University of Birmingham, Birmingham, UK; 2 Warwick Medical School, University of Warwick, Warwick, UK; 3 University Hospitals Birmingham NHS Foundation Trust, Birmingham, UK; 4 National FGM Centre, Barnardo's, Ilford, Essex, UK; 5 Birmingham Women's and Children's NHS Foundation Trust, Birmingham, UK; 6 Institute of Metabolism and Systems Research, University of Birmingham, Birmingham, UK; 7 School of Nursing, University of Birmingham, Birmingham, UK

**Keywords:** qualitative research, maternal medicine, gynaecology, public health, reproductive medicine

## Abstract

**Introduction:**

Female genital mutilation (FGM) is a significant global health concern and is likely to become an increasingly important healthcare challenge in destination countries such as the UK owing to rising levels of migration from FGM-affected countries. Currently, there is no consensus on the optimal timing of deinfibulation (opening) surgery for women who have experienced type 3 FGM and care provision remains suboptimal in the UK. This qualitative study aims to explore the views of survivors, male partners and healthcare professionals (HCPs) on the timing of deinfibulation and delivery of NHS FGM services.

**Methods and analysis:**

A qualitative study, informed by the Sound of Silence conceptual framework, will be undertaken via two work packages (WPs). WP1 will explore views on timing preferences for deinfibulation and NHS FGM services through interviews and discussion groups with FGM survivors (n~50), male partners (n~10) and HCPs (n~50). WP2 will use established techniques via two workshops (community (n~20–25 participants) and national stakeholder (n~30–35 participants)) to synthesise qualitative research findings and inform best practice and policy recommendations around the timing of deinfibulation and NHS FGM care provision. Supported by trained interpreters, data collection will be audio recorded and transcribed. Data will be analysed using the framework method to facilitate a systematic mapping and exploration of qualitative data from multiple sources.

**Ethics and dissemination:**

The study has received ethical approval from the North West Greater Manchester East Research Ethics Committee (18/NW/0498). The outputs for this study will be recommendations for best practice and policy around FGM care provision that reflects the views and preferences of key stakeholders. The findings will be disseminated via conference presentations, peer-reviewed publications, patient groups, third sector organisations and social media.

**Trial registration number:**

ISRCTN14710507.

**Strengths and limitations of this study**

- This is one of the largest qualitative studies in the UK to explore views on the timing of deinfibulation and NHS female genital mutilation (FGM) care provision with a range of key stakeholders including survivors, male partners and healthcare professionals (HCPs).
- This cross-cultural and cross-language study will allow us to hear narratives from a range of FGM-affected communities across the UK.
- This qualitative study is informed by the Sound of Silence conceptual framework.
- The study is supported by an active FGM survivor group, a charity partner, a number of NHS trusts and third sector organisations.
- Recruitment, data collection and interpretation may be challenging due to the cross-cultural and cross-language nature of the study.

## Introduction

Female genital mutilation or cutting (hereafter referred to as FGM) is an increasingly global issue owing to international mobility and migration.^1^ The practice of FGM has been performed for millennia^2^ and continues to be prevalent in some countries in Sub-Saharan Africa, Asia and the Middle East.^3^ An estimated 200 million women and girls live with FGM globally.^1^ In the UK, FGM is identified increasingly among migrants from FGM-affected countries, with 137 000 women and girls reported as currently living with the consequences.^4^ Between April 2015 and December 2017, 15 390 women and girls presented to NHS services where FGM was identified as a relevant condition and/or treated.^5^ The annual cost of NHS care for FGM survivors is estimated at £100 million.^6^

FGM involves the partial or complete removal of, or injury to, the external female genitalia for non-medical reasons.^7^ The WHO joint statement has classified FGM into four main types (types 1–4), with a further seven subtypes (types 1a,b; 2a–c; 3a,b) identified to capture more closely the variation in practices.^8 9^ Generally, the extent of genital tissue cut increases from type 1 to type 3, with type 3 (infibulation) being the most extensive and often requiring deinfibulation^7^ (a surgical procedure to release the narrowed vaginal introitus). There are immediate and lifelong health, obstetric, sexual functioning, psychosocial and economic impacts associated with FGM.^4 10–17^ The risks of adverse outcomes appears to be greater the more extensive the FGM,^15^ with 9 in 10 type 3 FGM survivors reporting complications.^18^ The consequences of type 3 FGM may lead to loss of life and reduced quality of life.^10 19^ The WHO reports^20^ that deinfibulation is associated with improved health and well-being, as well as, allowing sexual intercourse and childbirth, although there is limited direct evidence to support this statement. A recent systematic review found no evidence that deinfibulation improved urological complications.^21^ There is however a case to be made that deinfibulation is associated with improved gynaecologic and obstetric outcomes, although this is based on low-quality observational evidence.^22^

There has been slow progress in the development of evidence-based care to improve health outcomes for FGM survivors, in particular, around the optimal timing of deinfibulation.^20 23 24^ Deinfibulation can be undertaken outside of or during pregnancy^24^; however, there is considerable variation within and between clinical recommendations for when deinfibulation should occur.^23^ For example, Royal College of Obstetricians and Gynaecologists (RCOG) guidelines^25^ recommend that deinfibulation should be offered prior to pregnancy and preferably before first sexual intercourse. These guidelines also state that deinfibulation can be performed antenatally, in the first stage of labour, at delivery or perioperatively after a caesarean section. Royal College of Nursing (RCN) FGM guidance^26^ does not provide a clear indication on the optimal timing of deinfibulation, with one statement indicating that the procedure is best performed when not pregnant and another that deinfibulation is best undertaken before or at least within the second trimester of pregnancy. WHO guidelines on the management of FGM^20^ recommend either antepartum or intrapartum deinfibulation with a suggestion that timing should be based on wider contextual factors including patient preference, access to healthcare facilities, place of delivery and the skill level of the healthcare professional (HCP). In addition to a lack of consensus about when deinfibulation should be performed, there is also debate about whether timing affects outcomes, with some studies suggesting that obstetric risks increase the later deinfibulation is undertaken.^27 28^ However, these findings were not substantiated in a recent systemic review of low-quality observational evidence comparing childbirth outcomes between antepartum and intrapartum deinfibulation.^24^

A gap analysis of current FGM research has suggested that there is an urgent need for well-designed research to inform evidence-based guidelines and to improve the healthcare of women and girls with FGM.^23^ A recent qualitative systematic review highlighted that it is important for future research to focus on care-seeking and decision making around the timing of deinfibulation.^29^ To date, in the UK, there has been one qualitative study that directly explored women’s experiences of deinfibulation^30^ and no studies exploring the views of men and HCPs. With no clear consensus on the optimal timing of deinfibulation for type 3 FGM survivors,^20 23 24^ there is a specific need to focus on exploring preferences, involving a diverse range of FGM survivors, their male partners and HCPs, across multiple centres.^23 31^ Such research may help to inform the strategic planning and development of cost-effective, culturally acceptable NHS services, leading to improved outcomes for women and their families and help other organisations (eg, third sector organisations) plan for improved support for FGM survivors and their families. This paper presents the protocol for the FGM Sister Study.

### Aims and objectives

The aim of the FGM Sister Study is to explore and understand the views of FGM survivors, male partners and HCPs on the timing of deinfibulation and how NHS services can best be delivered to meet the needs of FGM survivors and their families. We will address this overarching aim via two work packages (WPs). The aims and objectives of each WP are presented in table 1.

**Table 1:** Summary of FGM Sister Study aims and objectives by work package

Work package 1	Work package 2
**Aim**: To qualitatively explore and understand the timing preferences for deinfibulation and how NHS FGM services could be improved for type 3 FGM survivors (WP1a), their male partners (WP1b) and HCPs (WP1c).	**Aim**: To use established techniques with survivors (WP2a) and stakeholders (WP2b) to synthesise the qualitative research findings, inform best practice and policy recommendations around the timing of deinfibulation and FGM care provision and identify future actions.
**Objectives**: to explore knowledge, awareness and understanding of FGM and deinfibulation (WP1a,b,c) to elicit views on preferences for the timing of deinfibulation and the rationale for these (WP1a,b,c) to explore perspectives on the decision making process around deinfibulation (WP1a,b) to explore knowledge, awareness and experiences of FGM services and support (WP1a,b,c) to understand the enablers, motivators and barriers to FGM care seeking behaviours (WP1a,b) to explore how HCPs describe, explain and reason about their care provision for FGM survivors and their families (WP1c) to understand how FGM care provision could be improved to best meet the needs of FGM survivors, their families and HCPs who support them in their local context (WP1a,b,c)	**Objectives**: to explore views and reflections on the trustworthiness of our interpretation of the data and the conclusions drawn (WP2a,b) to establish if there is consensus about the optimal timing of deinfibulation (WP2a,b) to identify the key recommendations to inform NHS FGM care provision (WP2a,b) to explore the facilitators and barriers to implementation of changes to NHS FGM care provision (WP2b) to explore views on the requirements for future FGM research (eg, RCTs) (WP2b)

FGM, female genital mutilation; HCPs, healthcare professionals; RCT, randomised clinical trial; WP, work package.

## Methods and analysis

### Study design and conceptual framework

The FGM Sister Study is a qualitative study, informed by the *Sound of ‘Silence’* (‘Silences’) conceptual framework.^32^ The ‘Silences’ framework is underpinned by broader theoretical approaches with a worldview that accepts that reality (or ‘truth’) is not objective, rather the social world is influenced by people in a particular society at a particular point in time.^33^ ‘Silences’ define areas of research and experiences that are little researched, understood or not heard^34^ and are useful for researching sensitive issues and/or the healthcare needs and perspectives of marginalised populations.^32^ Within the context of this study, although FGM is a contemporary issue that has increasingly become the subject of political and media interest, it remains a sensitive issue prevalent among marginalised populations and one that is under-researched. ‘Silences’ elucidates and underpins the research using a four-stage approach: (1) working in Silences, (2) hearing Silences, (3) voicing Silences and (4) working with Silences. There is an additional fifth stage, (5) planning for Silences, that is not incorporated into the core four-stage model but will be used to help inform service delivery action planning and future recommendations for research, policy and practice

### Study setting and timing

The study will be undertaken with FGM survivors (WP1a and WP2a) and male partners (WP1b) in three high FGM prevalence areas of England (Birmingham, Manchester and London) and with HCPs (WP1c) and key stakeholders (WP2b) across the UK including areas of high and low FGM prevalence. Data collection for WP1 commenced in September 2018, and initial analyses are planned to be completed ahead of the start of WP2 in November 2019. Synthesis of the results of WP1 and WP2 and final recommendations are planned to be completed by May 2020.

### Eligibility

#### WPs 1a, 1b and 2a

Women who have experienced FGM and male partners of women who have experienced FGM will be eligible to participate if they are aged 18 years or over, are resident in the UK, speak fluent English, Somali, Arabic or French and are willing and able to provide voluntary informed consent (written, electronically completed or verbally). We will exclude anyone where their clinician, support worker or the researcher judges that their distress relating to FGM may influence their ability to provide voluntary informed consent. Additionally, we will not snowball recruitment of a male partner where the participating FGM survivor does not consent to their participation.

#### Work package 1c

HCPs (including but not limited to general practitioners, practice nurses, midwives, obstetrics and gynaecology clinicians, genitourinary clinicians, health visitors and sexual health specialists) will be eligible to participate if they are aged 18 years or over, speak fluent English, are willing and able to provide written, electronically completed or (audio recorded) verbal informed consent and are currently or have recently been involved (within the last 5 years) in the delivery of care to FGM survivors and their families in the UK.

#### Work package 2b

Key FGM stakeholders including (but not limited to) HCPs (see list in WP1c eligibility), policy makers, FGM specialist researchers/academics, health economists, commissioners and representatives from third sector organisations (eg, charities and advocacy groups) will be eligible to participate if they are aged 18 years and over, speak fluent English, are willing and able to provide written, electronically completed or (audio recorded) verbal informed consent and are currently or have recently been involved (within the last 5 years) in some aspect of service and/or care provision to FGM survivors and their families in the UK.

### Sampling and recruitment

#### Work package 1a

Four groups of pregnant and non-pregnant FGM survivors will be purposively sampled^35^ including those: (1) who have not had deinfibulation, (2) who have had deinfibulation for health and/or personal reasons, (3) who had deinfibulation antenatally and (4) who had deinfibulation during labour/at the point of delivery (intrapartum). Within these groups, we will try to ensure that we have maximum variation and diversity of views by including women from a range of FGM-affected communities (eg, Somali, Yemeni, Eritrean), locations, ages and education levels.^36^ Women will be recruited via multiple pathways including, for example, their HCP; advertising within FGM clinics, community settings and on social media, culturally sensitive snowballing^37^ from women approached to participate and FGM community groups/third sector organisations. Multiple NHS trusts and third sector organisations (eg, charities and advocacy groups) will support the study. Relevant staff at participant identification sites will be trained by the research team (via a site initiation visit or equivalent) to approach potential FGM survivors. Women will be approached, in the first instance, by a member of their usual care team (eg, midwife) or by a trusted advocate in a third sector organisation. Recruiters will be asked to briefly screen for eligibility and introduce the study. All documents to support participant identification and recruitment will be available in English, French, Somali and Arabic. If the woman responds positively, she will be asked to complete and sign a contact details form giving permission to be contacted by the research team. A researcher will then contact the woman to check her eligibility, discuss the study further, answer any questions she might have and arrange a mutually convenient time and location for the interview or to let her know the times and locations of discussion groups. At the end of the interview/discussion group, women will be asked discretely if their partner may wish to participate in the study. If she responds positively, then contact details of the research team will be left or if the man is present, then the recruitment and consent process will be initiated.

#### Work package 1b

Men will be identified via participants from WP1a, via support of local community groups and third sector organisations and via social media.

#### Work package 1c

HCPs will be purposively recruited^35^ from across a range of groups including (but not limited to) general practitioners, practice nurses, midwives, obstetrics and gynaecology clinicians, genitourinary clinicians, health visitors and sexual health specialists. HCPs across the UK will be identified via multiple pathways including, for example via: FGM service listings; contacting NHS trusts with maternity services in low FGM prevalence areas directly; the study team’s FGM networks; advertising the study via electronic communications (eg, social media), professional bodies and membership societies; and snowballing from HCPs approached to participate. HCPs who express an interest in participating will be screened for eligibility by the research team, provided with further study information, given time to answer questions and then a mutually convenient time and location for the interview arranged, or they will be told about the times and locations of discussion groups.

#### Work package 2a

FGM survivors who take part in WP1a will be invited to participate. If required, recruitment will be supplemented via the same pathways as identified in WP1a above.

#### Work package 2b

HCPs interviewed in WP1c will be invited to participate. Other key stakeholders (see list in WP1c and WP2b eligibility) will be identified via the research team’s networks and collaborators, social media and knowledge of FGM services acquired during the study.

### Anticipated sample sizes

#### WP1 (total n up to 110)

We will seek to recruit:

- up to 50 women who are FGM survivors,
- up to 10 male partners,
- up to 50 HCPs.

#### WP2 (total n up to 60)

We will seek to recruit:

- 20–25 FGM survivors for the community engagement event,
- 30–35 stakeholders for the national stakeholder event.

Numbers will however remain flexible to ensure that we collect sufficiently rich data to answer the research questions and achieve core analytic saturation.^38^

### Data collection

#### Work package 1

Semistructured interviews have been identified as an appropriate data collection method, given that they can facilitate an in-depth exploration of participants’ ‘Silent’ views^39^ and are particularly useful in discussions of sensitive or traumatic experiences such as FGM. In addition, discussion groups will be used as an alternative data collection tool for FGM survivors and male partners, as Patient and Public Involvement representatives (hereafter referred to as ‘the survivor group’) felt that women and men in some communities may be more willing, given the nature of the topic, to participate in a group rather than an individual discussion.

Interviews and discussion groups will be conducted by a trained qualitative researcher and two researchers will be present in all discussion groups. Independent, professional interpreters (who have undergone FGM training by the research team) will be employed to provide real-time oral translation (either face-to-face or on the phone) during the interviews/discussion groups where there is a language barrier between researcher(s) and participant(s). Debriefs between researcher(s) and interpreter(s) will be held after interviews/discussion groups if interpretation issues are identified. In addition, researchers will keep a research journal throughout the data collection period to help to provide reference points in the journey to expose ‘Silences’.^32^

Participants will be given the choice as to whether they wish to take part in an interview or a discussion group. If they choose to take part in an interview, they will be given a choice as to where it takes place, for example, in their own home, in a clinic room where they were recruited/work or via telephone (including Skype or other online communication tools). Discussion groups will take place in an appropriate location, for example, a community venue or clinic. Aligned with ‘Hearing Silences’ (stage 2 of the ‘Silences’ conceptual framework),^32^ discussion guides for WP1a–c will be informed by a critical reflection of the FGM evidence base (see online supplementary files 1–3). These ‘Silences’ will then be heard by and discussed within the research team and with the survivor group. Semistructured interviews and discussion groups will be conducted in a participant-focused manner, allowing experiences and views important to participants to develop naturally.^40^ The composition of the participants in each discussion group will be carefully considered, taking into account language, the community from which they are from, their deinfibulation experience and wider demographic characteristics.

Discussion guides will be refined iteratively to ensure that all views are captured. Data collection and analysis will take place concurrently^39^ and will continue until the research team judge that the data and sample have sufficient depth and breadth to address the research objectives.^38^ At (or before) each interview/discussion group, participants will be asked to complete a short demographic questionnaire to facilitate maximum variation sampling and provide a description of the sample characteristics.

#### Work package 2

A community engagement event (WP2a) and a national stakeholder (WP2b) event will be run by FGM experts at Barnardo’s, with support from the wider research team and the survivor group. Participants will be shown a plain English summary (drafted and discussed with the survivor group) in advance. At the start of each event, a study overview will be presented. Participants may be split into smaller discussion groups facilitated by a member of Barnardo’s and supported by a member of the research team and/or the survivor group. Discussion groups were identified as an appropriate data collection method, given that they provide an opportunity for interaction and communication between participants in order to generate data and can provide a permissive and empowering environment where participants feel comfortable enough to share their views and question those of others.^41–43^ Discussion will focus on participants’ reflections of the trustworthiness of our interpretation of the data and the conclusions drawn; an exploration of ‘what has or can change as a result of this study’ (aligned with Stage 4 ‘working with Silences’ of the ‘Silences’ conceptual framework^32^) in terms of NHS policy and practice and identifying future research to address other identified ‘unheard Silences’.^32^ Recommendations from each group will be shared and discussed within the whole group to establish if a consensus on the timing of deinfibulation can be reached and to identify the next steps following study completion.

### Data analysis

Interviews, discussion groups and events will be audio recorded and transcribed using an intelligent transcription system (ie, the conversation is transcribed verbatim but without unnecessary verbiage or linguistic fillers) by a specialist company and subsequently checked for quality and anonymised by the research team. Up to six transcripts of interpreted interviews will have both the English and the second language translated and transcribed. This is to enable the research team to check that interpreters are employing content interpretation rather than word-for-word translation and to ensure that interpreted responses are conducive to and reflect the depth of response provided by the participant.

Data analysis will be informed by framework analysis,^44^ which provides a systematic and flexible model for managing and mapping qualitative data from multiple sources. The research team will use a five-stage framework approach^45^ that captures, but also condenses, other framework approaches involving a greater number of stages. These five stages include (1) compiling (researchers collect data), (2) disassembling (researchers become familiar with and organise the data), (3) reassembling (researchers produce matrices to analyse the data), (4) interpreting (researchers interpret the data to identify key themes, concepts, relationships, etc) and (5) concluding (researchers present findings). Each stage of the framework approach can be aligned with the stages of the Sound of ‘Silence*’* conceptual framework to ensure relevant synthesis of qualitative methodology and philosophical/conceptual underpinnings that is sensitive to the research topic and is appropriate for the research study (see online supplementary file 4).

10.1136/bmjopen-2019-034140.supp4 Supplementary data

### Patient and public involvement

In addition to our co-applicant and Study Steering Group (SSG) FGM survivors, a lay group of type 3 FGM survivors, with different experiences of deinfibulation, has been established. Discussions, where possible, are co-facilitated by the chief investigator and our FGM survivor co-applicant. The survivor group and co-applicant have been actively involved in the design of the study and writing of the study documentation. For example, the group chose the logo and name of the study, have provided critical review of all participant facing materials and helped to co-produce the discussion guides. In addition, they will provide on-going problem solving support, critical review of interview transcripts and the research team’s interpretation of data and support dissemination plans.

## Ethics and dissemination

### Data management, monitoring and oversight

The University of Birmingham is the nominated sponsor and data controller for the study. Data management and storage will be subject to the UK Data Protection Act 2018 and will follow relevant University of Birmingham policy and procedures. Identifiable data will be securely stored and then safely destroyed within 12 months of publication of the main results of the study. Anonymised data will be securely stored for a minimum of 10 years after the publication of the main study results. An independently chaired SSG has been convened to oversee the study and includes an FGM survivor, academics, FGM-specialist HCPs and third sector representatives. The SSG has agreed the study protocol and will agree any subsequent amendments. This group will monitor adherence to protocol and participant safety and will ensure that the study runs in accordance with the principles of good clinical practice and relevant regulations.

### Outputs and dissemination

Dissemination is likely to focus on: the findings of the qualitative research with FGM survivors and their male partners; the qualitative research with HCPs working with FGM survivors and their families and the overarching policy and practice implications and recommendations of the research. The study final report (including a plain English summary co-produced with the survivor group) will be available through the National Institute for Health Research (NIHR) website, published in open access peer-reviewed journals and presented at relevant conferences and events.

10.1136/bmjopen-2019-034140.supp1 Supplementary data

10.1136/bmjopen-2019-034140.supp2 Supplementary data

10.1136/bmjopen-2019-034140.supp3 Supplementary data

## Supplementary Material

Reviewer comments
