# Supplementary material for: Exploring the views of female genital mutilation survivors, their male partners and healthcare professionals on the timing of deinfibulation surgery and NHS FGM care provision (the FGM Sister Study): protocol for a qualitative study
Source: BMJ Open. 2019 Oct 17;9(10):e034140. doi: 10.1136/bmjopen-2019-034140 (PMC6803147; doi:10.1136/bmjopen-2019-034140)
Supplement: Supplementary data [file bmjopen-2019-034140supp004.pdf]

Supplementary file 4: Alignment of the Framework Approach[1] with the stages of the Sound of 'Silence' conceptual framework[2] and our approach to data collection and analysis

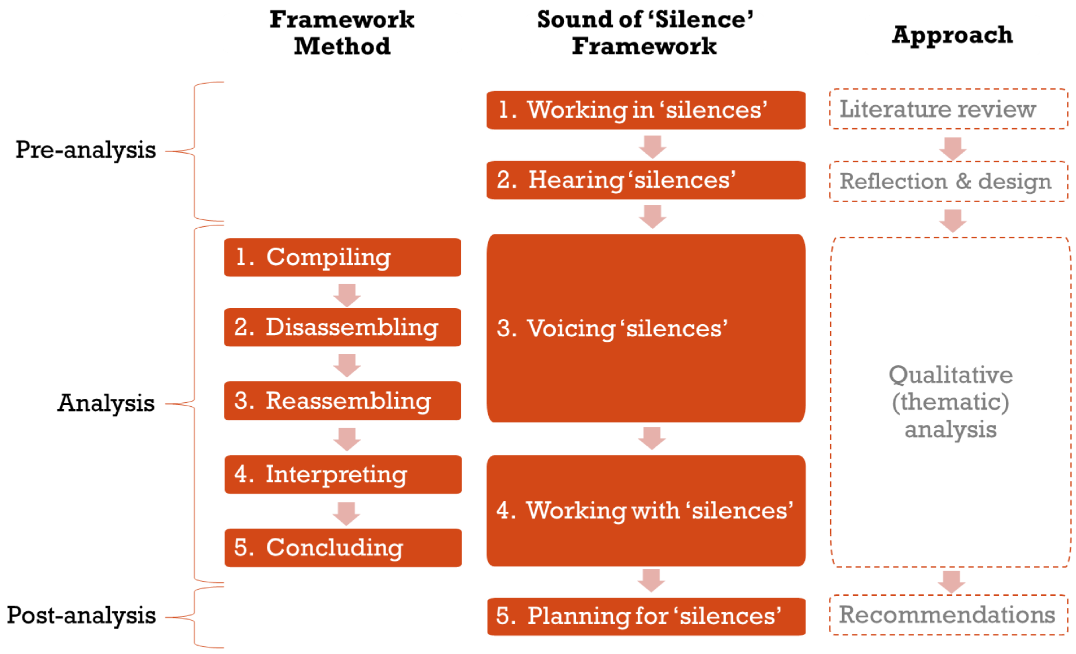

Supplementary file 4 references adapted from:

[1] Castleberry A, Nolen A. Thematic analysis of qualitative research data: Is it as easy as it sounds? *Curr Pharm Teach Learn* 2018;10(6):807-15. doi: 10.1016/j.cptl.2018.03.019 [published Online First: 2018/07/22]

[2] Serrant-Green L. The sound of 'silence': a framework for researching sensitive issues or marginalised perspectives in health. *Journal of Research Nursing* 2011;16(4)

Jones L, *et al. BMJ Open* 2019; 9:e034140. doi: 10.1136/bmjopen-2019-034140
