# Supplementary material for: Exploring the views of female genital mutilation survivors, their male partners and healthcare professionals on the timing of deinfibulation surgery and NHS FGM care provision (the FGM Sister Study): protocol for a qualitative study
Source: BMJ Open. 2019 Oct 17;9(10):e034140. doi: 10.1136/bmjopen-2019-034140 (PMC6803147; doi:10.1136/bmjopen-2019-034140)
Supplement: Supplementary data [file bmjopen-2019-034140supp002.pdf]

Supplementary file 2: Male Partners of Female Genital Mutilation Survivors Discussion Guide (Work Package 1b)

Focus	*Objective(s)* and key discussion points (nb. these are not direct questions)
FGM	*1a. to explore knowledge, awareness and understanding of FGM and deinfibulation*
	Explore preferred terminology around FGM<br>Explore understanding of FGM<br>Explore culture of and attitudes around FGM within their community/home country<br>Explore culture of and attitudes around FGM within the UK/receiving county (including FGM law in the UK/protection orders)<br>Explore personal experiences and impacts of FGM<br>Explore views, experiences and impacts of FGM on others (e.g. partner, wider family)
Deinfibulation	*1a. to explore knowledge, awareness and understanding of ~~FGM and~~ deinfibulation*
	1b. to elicit views on preferences for the timing of deinfibulation and the rationale for these
	1c. to explore perspectives on the decision making process around deinfibulation
	Explore preferred terminology around re-opening surgery (deinfibulation)<br>Explore cultural meaning of re-opening surgery (deinfibulation) within their community<br>Explore their views on re-opening surgery (deinfibulation)<br>Explore personal experiences of re-opening surgery (deinfibulation)<br>Explore personal decision making process around re-opening surgery (deinfibulation)<br>Explore preferences for timing of re-opening surgery (deinfibulation) (e.g. when, where, who how)
FGM-related care seeking	*1d. to explore knowledge, awareness, and experiences of FGM services and support*
	1e. to understand the enablers, motivators and barriers to FGM care seeking behaviours

Jones L, *et al. BMJ Open* 2019; 9:e034140. doi: 10.1136/bmjopen-2019-034140

	Explore awareness and any experiences of accessing FGM-related care (including re-opening surgery (deinfibulation)) in home country (for themselves and/or their partner)<br>Explore awareness and any experiences of accessing FGM-related care (including re-opening surgery (deinfibulation)) in UK/receiving country (for themselves and/or their partner)<br>Explore awareness of local (UK) FGM services<br>Explore enablers/motivators for accessing FGM-related care for themselves and/or their partner (including re-opening surgery (deinfibulation))<br>Explore barriers to accessing FGM-related care for themselves and/or their partner (including re-opening surgery (deinfibulation))
Future NHS FGM-related care provision	*1g. to understand how FGM care services could be improved to best meet the needs of FGM-survivors, their families and HCPs who support them in their local context*
	Explore what services/support/interventions should be offered to male partners and FGM-survivors (e.g. what, where, who)<br>Explore how current local FGM services could be improved<br>Explore what services can do to support male partners and FGM-survivors

FGM: Female Genital Mutilation

Jones L, *et al. BMJ Open* 2019; 9:e034140. doi: 10.1136/bmjopen-2019-034140
