# Supplementary material for: Exploring the views of female genital mutilation survivors, their male partners and healthcare professionals on the timing of deinfibulation surgery and NHS FGM care provision (the FGM Sister Study): protocol for a qualitative study
Source: BMJ Open. 2019 Oct 17;9(10):e034140. doi: 10.1136/bmjopen-2019-034140 (PMC6803147; doi:10.1136/bmjopen-2019-034140)
Supplement: Supplementary data [file bmjopen-2019-034140supp003.pdf]

Supplementary file 3: Healthcare Professionals Discussion Guide (Work Package 1c)

Focus	*Objective(s)* and key discussion points (nb. these are not direct questions)
FGM	*1a. to explore knowledge, awareness and understanding of FGM and deinfibulation*
	Explore awareness and knowledge of terminology around FGM<br>Explore understanding of FGM and FGM types<br>Explore understanding of culture and attitudes around FGM within the UK and FGM-affected countries)<br>Explore understanding of impacts/consequences of FGM for survivors and wider family members (e.g. male partners and children)<br>Explore awareness around and attitudes towards FGM law in the UK/protection orders (including influence on practice)<br>Explore awareness and knowledge of local, national, international FGM guidelines/policies
Deinfibulation	*1a. to explore knowledge, awareness and understanding of FGM and deinfibulation*
	1b. to elicit views on preferences for the timing of deinfibulation and the rationale for these
	Explore awareness and knowledge of terminology around deinfibulation<br>Explore understanding of cultural meaning of deinfibulation within UK and FGM-affected communities<br>Explore their personal views on deinfibulation<br>Explore understanding of the outcomes of deinfibulation for FGM-survivors (and their male partners)<br>Explore preferences for timing of deinfibulation (e.g. when, where, who how)
FGM-related care provision	*1d. to explore knowledge, awareness, and experiences of FGM services and support*
	1e. to understand the enablers, motivators and barriers to FGM care seeking behaviours
	1f. to explore how HCPs describe, explain and reason about their care provision for FGM-survivors and their families

Jones L, *et al. BMJ Open* 2019; 9:e034140. doi: 10.1136/bmjopen-2019-034140

	Explore experiences of providing FGM-related care (including deinfibulation)<br>Explore awareness of local (UK) FGM-related services (outside of their own experiences including NHS and non-NHS<br>Explore views on current UK services/support/interventions offered to FGM-survivors and wider family (e.g. what, where, who)<br>Explore perceptions around the enablers/motivators for survivors and their families to accessing FGM-related care (including deinfibulation)<br>Explore perceptions around the barriers for survivors and their families to accessing FGM-related care (including deinfibulation)
Future NHS FGM-related care provision	*1g. to understand how FGM care services could be improved to best meet the needs of FGM-survivors, their families and HCPs who support them in their local context*
	Explore thoughts on what services/support/interventions should be offered to FGM-survivors and their wider family (e.g. what, where, who)<br>Explore how current UK FGM services could be improved<br>Explore views on whether the UK FGM Law/Protection Orders needs to be changed<br>Explore what services can do to support FGM-survivors and their wider family

FGM: Female Genital Mutilation; HCP: Healthcare Professional
