# Supplementary material for: Exploring the views of female genital mutilation survivors, their male partners and healthcare professionals on the timing of deinfibulation surgery and NHS FGM care provision (the FGM Sister Study): protocol for a qualitative study
Source: BMJ Open. 2019 Oct 17;9(10):e034140. doi: 10.1136/bmjopen-2019-034140 (PMC6803147; doi:10.1136/bmjopen-2019-034140)
Supplement: Reviewer comments [file bmjopen-2019-034140.reviewer_comments.pdf]

PEER REVIEW HISTORY

BMJ Open publishes all reviews undertaken for accepted manuscripts. Reviewers are asked to complete a checklist review form (**http://bmjopen.bmj.com/site/about/resources/checklist.pdf**) and are provided with free text boxes to elaborate on their assessment. These free text comments are reproduced below.

ARTICLE DETAILS

**TITLE (PROVISIONAL)**	Exploring the views of female genital mutilation survivors, their male partners and healthcare professionals on the timing of deinfibulation surgery and NHS FGM care provision (the FGM Sister Study): protocol for a qualitative study
**AUTHORS**	Jones, Laura; Danks, Emma; Clarke, Joanne; Alidu, Lailah; Costello, Benjamin; Jolly, Kate; Byrne, Alison; Fassam-Wright, Meg; Latthe, Pallavi; Taylor, Julie

This article was not externally reviewed at BMJ Open. Protocols that have been independently assessed prior to submission to BMJ Open are usually fast-tracked to publication on the grounds that further substantial changes will not be possible. This independent assessment will usually be external, independent review for both a substantial grant award from a non-commercial or government funder and ethics approval.
